# Statistical Parametric Mapping reveals sex-specific, phase-dependent differences in bilateral three-dimensional ground reaction force during Wushu Changbing PiJi in high-level athletes

**DOI:** 10.1371/journal.pone.0359797

**Published:** 2026-11-04

**Authors:** Jinyong Feng, Lianzhen Ma, Yupeng Shen, Shuxin Wang, Yiyi Qiu, Pengfei Zheng

**Affiliations:** 1 Sports Engineering Center, School of Physical Education and Sports Science, South China Normal University, Guangzhou, China; 2 School of Physical Education and Health, Guangdong Polytechnic Normal University, Guangzhou, China; Somaiya Vidyavihar University K J Somaiya College of Engineering, INDIA

## Abstract

PiJi is a core attacking technique in Wushu Changbing competition, but continuous waveform evidence remains limited regarding the sex-specific phase characteristics of bilateral 3D ground reaction force (GRF) during PiJi performed with maximal voluntary effort (MVE). This study aimed to compare continuous changes in trailing-foot, lead-foot, and net bilateral GRF between high-level male and female athletes during PiJi performed with MVE. In this cross-sectional, laboratory-based observational study, 44 right-hand-dominant athletes, including 22 male athletes and 22 female athletes, performed PiJi with MVE, yielding 616 valid trials. Bilateral 3D GRF was recorded using two force plates. Each trial was time-normalized and linearly interpolated to 143 equally spaced nodes, and within-participant mean waveforms were calculated before between-group differences were analyzed using statistical parametric mapping. Primary analyses used random-field-theory inference, with sensitivity examined using 5,000 permutation tests. After Bonferroni correction, phase-specific sex differences were detected in trailing-foot, lead-foot, and net bilateral 3D GRF. Scalar localization showed that male athletes had higher trailing-foot $F_{y}$ during the middle phase (33.98%–47.04%) and higher trailing-foot $F_{x}$ (62.41%–71.99%) and net bilateral $F_{x}$ (54.18%–70.41%) during the middle-to-late phase; female athletes showed only a brief period of higher trailing-foot vertical $F_{z}$ during the middle phase (42.11%–44.61%). Although lead-foot 3D GRF differed overall, no confirmatory difference was localized to any single scalar component. These findings indicate that sex-related GRF differences in high-level PiJi were concentrated mainly in specific horizontal-plane phases of trailing-foot and net bilateral force, whereas the lead-foot difference was identified only at the overall 3D GRF level. These results provide kinetic evidence for phase-specific assessment of PiJi technique and suggest that training monitoring and mechanistic research should consider foot, direction, and movement phase rather than relying only on peak force or discrete variables. Future studies integrating multimodal measures and sport-specific performance may further clarify the functional significance of bilateral force-generation patterns.

## 1. Background

Wushu Changbing is an armed combat discipline that uses the long staff as its primary implement and inherits the Ming-dynasty Shaolin Fengmogun routine, which is currently preserved mainly through the Ma-style Tongbei Wushu school [1,2]. PiJi is the most representative attacking technique in Changbing and consists of a preparatory backswing for energy storage followed by an explosive downward strike. Its primary objective is to achieve rapid acceleration of the weapon tip and high kinetic energy output within a short time window [1,2]. Because the force, speed, and impact stability of PiJi are directly related to sport-specific performance, clarifying its underlying force-generation mechanism is of considerable importance. Studies on comparable implement-based striking tasks have shown that ground reaction force (GRF) is closely associated with endpoint velocity, technical level, and striking performance [3,4]. However, during maximal-effort PiJi, how the two feet generate, distribute, and coordinate 3D GRF remains insufficiently supported by clear kinetic evidence.

This issue is central because a striking movement performed with relatively fixed foot positions does not rely solely on overall force production. Instead, it depends on the functional differentiation of the lead-foot and trailing-foot during propulsion, braking, rotational drive, and stability control. Research on golf swing mechanics has shown that bilateral GRF patterns and center-of-pressure shifts are associated with clubhead speed and technical level, with skilled athletes generally demonstrating more effective load transfer between the lead-foot and trailing-foot [3]. In baseball batting, vertical and posterior GRF of the lead-foot are related to bat speed at ball contact [4], whereas horizontal GRF is associated with trunk mechanical energy flow, suggesting that the efficiency of force transfer from the lower limbs to the trunk and implement may be related to the direction and timing of bilateral foot loading [5]. Kendo striking research has similarly shown that different striking tasks exhibit distinct lower-limb joint moment and GRF timing characteristics [6]. Therefore, existing evidence suggests that the phase-specific characteristics of external GRF during striking are related to technical performance and whole-body kinematics, but their specific relationship in PiJi remains to be verified.

Methodologically, traditional discrete biomechanical variables are insufficient to fully characterize this process. Although discrete variables are easy to interpret, they compress the continuous force-generation process into a limited number of feature points and may therefore miss local differences within specific movement phases, particularly limiting the identification of phase-specific force-generation strategies during rapid explosive movements [7,8]. In contrast, continuous GRF waveforms preserve information on force magnitude, direction, and timing throughout the movement cycle, thereby providing a more detailed representation of time-dependent differences that are difficult to capture using traditional discrete statistics. Existing studies have shown that continuous waveform approaches can more accurately characterize the relationship between technical features and movement performance [9,10]. Statistical Parametric Mapping (SPM) is a random-field-theory-based statistical method for continuous data that enables global testing of one-dimensional or multidimensional biomechanical curves over a normalized movement cycle and identifies the specific time intervals in which significant differences occur [11,12]. In recent years, SPM has been applied to temporal analyses of running GRF [13], kinetic analyses of the golf swing [14], GRF time-series analyses in baseball pitching [15], and GRF vector-field analyses in sprinting [16], demonstrating its advantages in preserving waveform integrity, controlling multiple-comparison error, and interpreting phase-specific biomechanical differences. Therefore, SPM is well suited to analyzing the continuous changes in bilateral three-dimensional GRF during PiJi and to identifying force-generation differences between high-level male and female athletes across different movement phases.

The functional differentiation of the two feet may be further shaped by sex differences in knee-flexor and knee-extensor morphology, which may contribute to differences in lower-limb force-production capacity and muscular balance [17]. Pelvic-hip geometry can also alter hip-abductor mechanics and the moment arm of mediolateral GRF, providing an anatomical basis for sex-specific force-vector regulation [18]. In fixed-stance batting, coordinated hip and knee actions generate bilateral mediolateral GRF that contributes to whole-body rotation [19], while horizontal GRF is associated with mechanical-energy inflow from the hip into the lower torso [5]. Because the same standardized Changbing imposes different relative task demands according to body size, we expected sex differences in PiJi GRF to manifest not simply as greater magnitudes in male athletes but as foot-, direction-, and phase-specific force-organization patterns [20].

This study used SPM to analyze the continuous characteristics of bilateral 3D GRF in high-level Wushu Changbing athletes during PiJi performed with maximal voluntary effort (MVE). The aim was to identify sex-specific differences and phase-specific GRF characteristics of the lead-foot, trailing-foot, and net bilateral GRF across different portions of the movement cycle. Based on existing evidence that the temporal characteristics of external GRF from the lead-foot and trailing-foot may be functionally differentiated in implement-based striking tasks [3,21,22], this study hypothesized that, during PiJi performed with MVE, bilateral 3D GRF would exhibit movement-cycle-specific sex differences between high-level male and female athletes.

## 2. Materials and methods

### 2.1. Participants

This study employed a cross-sectional, laboratory-based observational design to compare bilateral GRF waveforms between male and female athletes; although the PiJi task was standardized, no intervention or random allocation was applied. Participants were recruited prospectively using a non-probability, criterion-based purposive sampling approach. Potential participants were screened against the prespecified eligibility criteria described below before enrollment. A total of 50 Wushu Changbing athletes were recruited for this study, including 25 male athletes and 25 female athletes. All participants were right-hand dominant, had more than 2 years of specialized training experience, had achieved a top-three ranking at least once in a national collegiate Wushu Changbing competition within the previous 2 years, and had no history of lower-limb orthopedic surgery or musculoskeletal injury affecting movement execution within the 6 months before testing. Before testing, individuals with acute pain, fatigue, or medication use that could affect motor performance were excluded; testing for female participants was scheduled during the early follicular phase. After data-quality screening, three male and three female athletes were excluded because of excessive between-trial variability and non-standardized PiJi execution, resulting in a final analytical sample of 44 athletes. Based on an effect-size sensitivity analysis conducted in G*Power 3.1.9.7, under the conditions of a two-tailed independent-samples test, α=0.05, statistical power of 1−β=0.80, and a group allocation ratio of 1, the minimum detectable effect size for the current sample was approximately Cohen’s d = 0.865. The effect-size calculation was a sensitivity analysis for the final analytical sample and was not used to determine recruitment targets or to provide a formal power estimate for the SPM analyses.

Participants were prospectively recruited and tested between 27 February 2026 and 26 April 2026. The study protocol was reviewed and approved by the Ethics Committee of South China Normal University (approval no. SCNU-SPT-2025–033). All experimental procedures complied with the Declaration of Helsinki. No minors were included in this study. All participants voluntarily provided written informed consent after being fully informed of the study purpose, experimental procedures, and potential risks. Based on their top-three placing in a national collegiate Wushu Changbing competition within the previous 2 years, the cohort was retrospectively classified as Tier 3 (Highly Trained/National Level) according to the Participant Classification Framework proposed by McKay et al. (2022) [23]. This cohort-level classification was based on the available national-level performance information; weekly training volume and periodization were not recorded, and no international performance metric was documented to support Tier 4 classification.

### 2.2. Experimental protocol

Before formal data collection, all participants completed a standardized 25-min warm-up (Fig 1, stage 1). Familiarization was conducted immediately before formal testing during the PiJi movement stage (Fig 1, stage 3), rather than in a separate session. Both the number of practice trials and the familiarization duration were self-selected and were not recorded. Formal testing began only after each participant confirmed that they were fully warmed up and ready to perform PiJi with MVE. During formal testing, participants performed 14 technically standardized PiJi trials simulating target striking with MVE [24,25]. To accommodate individual recovery requirements and minimize potential fatigue during the repeated MVE trials, the rest interval between consecutive trials was self-selected rather than fixed, an approach previously used in repeated maximal-performance and maximal-kicking protocols [26,27]. Each subsequent trial began only after the participant reported being ready to perform another maximal-effort PiJi. Rest interval durations were not recorded.

**Fig 1:**
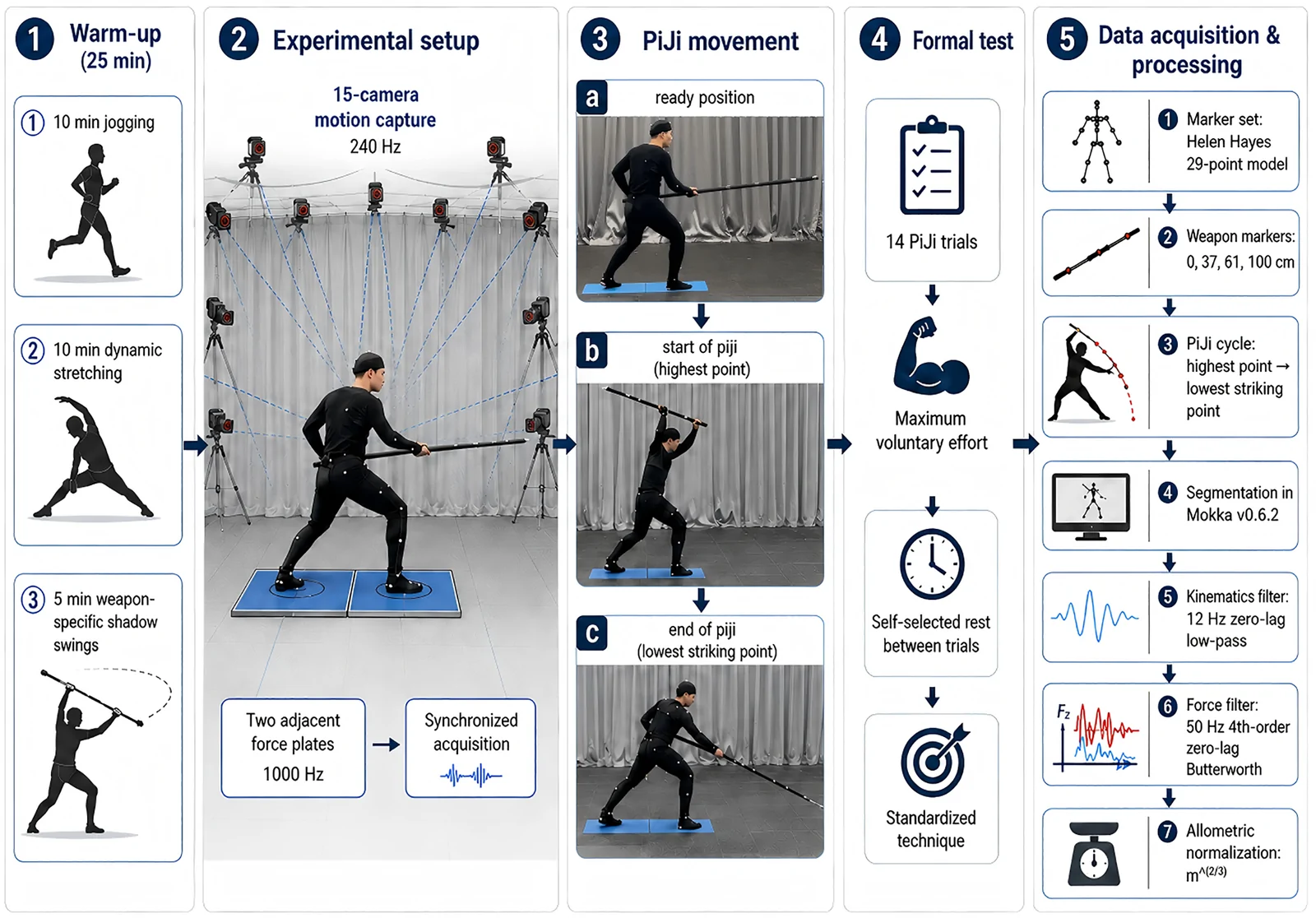
Experimental protocol flowchart. The experimental protocol comprised five stages: (1) a standardized 25-min warm-up; (2) experimental setup and synchronization of the motion-capture and force-plate systems; (3) familiarization and execution of the PiJi movement from the preparatory position to the lowest striking point; (4) 14 maximal-effort PiJi trials with self-selected rest intervals; and (5) data acquisition and processing. This original figure was created by Pengfei Zheng and is published under a CC BY 4.0 license with permission from the copyright holder, Pengfei Zheng; original copyright 2026.

### 2.3. Data acquisition and processing

During data acquisition, participants held a standard Changbing (length: 2.0 m; mass: 1.0 kg) with both hands while adopting a preparatory stance with the right foot forward and the left foot backward (Fig 1, panel 3a). During testing, all athletes wore standard Wushu shoes routinely used during training and competition, rather than performing barefoot. Although the preparatory configuration and PiJi technique were standardized, stance width, step length, and foot angle were not prescribed using fixed values or anthropometric scaling; instead, these parameters were self-selected within the technical requirements to preserve each athlete’s habitual execution. Each foot was placed independently in the central region of one of two adjacent 3D force plates (Kunwei Technology, Jiangsu, China; sampling frequency: 1000 Hz). The force plates were embedded flush with the surrounding hard floor and were left uncovered. No carpet or mat was placed over the force plates to avoid introducing an additional interface that could alter force transmission. The original force-plate surfaces had an integral slip-resistant finish suitable for dynamic movement testing.

The force plates were temporally synchronized with a 15-camera 3D motion capture system (Nokov, Beijing, China; sampling frequency: 240 Hz). Retroreflective markers were placed on the body according to the Helen Hayes 29-marker model to assist movement-phase identification. Four markers were fixed along the longitudinal axis of the Changbing implement at 0, 37, 61, and 100 cm from the bottom end to reconstruct its 3D trajectory. The marker located 100 cm from the bottom end was designated M4 and was used to identify the lowest striking point. The M4 vertical-coordinate trajectory was processed using a 12-Hz zero-phase low-pass filter. The lowest striking point was operationally defined as the first local minimum in the filtered vertical coordinate (+Z) of M4 after the highest-point event during each trial. This candidate event was automatically identified and then verified in Mokka by two assessors. No fixed absolute height or vertical-velocity threshold was applied because the absolute terminal position varied among athletes; instead, the same within-trial criterion was used to accommodate individual differences in PiJi execution.

The outputs from both force plates were expressed in the same fixed laboratory coordinate system: the X-axis was the mediolateral axis, with +X pointing to the participant’s left side; the Y-axis was the anteroposterior axis, with +Y pointing posteriorly; and the Z-axis was the vertical axis, with +Z pointing upward. In the experiment, the right front foot was defined as the lead-foot, the left rear foot as the trailing-foot, and the resultant force from both feet as the net bilateral GRF. To effectively reduce signal noise, raw motion-capture and force-plate data were smoothed using a 12-Hz zero-phase low-pass filter and a 50-Hz fourth-order zero-lag Butterworth low-pass filter, respectively [28,29]. Body mass and body-fat percentage were measured concurrently using a HUAWEI Scale 3 Pro bioelectrical impedance scale (Huawei, Shenzhen, China). Lean body mass (m) was calculated as $body\;mass\;\times \;\left(\text{1}\;-\;\frac{body\;fat\;percentage}{\text{1}\text{0}\text{0}}\right)$. All GRF components were normalized allometrically to lean body mass raised to the power of 2/3 (${\text{m}}^{\text{2}/\text{3}}$); specifically, each force value was divided by ${\text{m}}^{\text{2}/\text{3}}$.

### 2.4. Data analysis

All PiJi trials were first mapped to a complete movement cycle from 0% to 100%. When multiple original sampling points were mapped to the same normalized time point, the corresponding force values were averaged at that position. The $F_{x}$, $F_{y}$, and $F_{z}$ components of the trailing-foot, lead-foot, and net bilateral GRF were then resampled to 143 equally spaced nodes using linear interpolation. For multiple trials from the same athlete, time normalization and interpolation were first performed separately for each trial, after which the within-participant mean waveform was calculated and used as the independent observational unit for between-group statistical analysis.

Between-sex differences in GRF waveforms across the entire movement cycle were inferred using one-dimensional SPM based on random field theory. For each continuous waveform, SPM controlled the family-wise error rate across the 143 time nodes; for multiple parallel tests across GRF waveforms, a prespecified Bonferroni correction was further applied. As the primary confirmatory analysis, 3D GRF vector fields were defined by the $F_{x}$, $F_{y}$, and $F_{z}$ components separately for the trailing-foot, lead-foot, and net bilateral GRF. Two-sample SPM Hotelling’s $T^{\text{2}}$ tests were then used to evaluate overall 3D waveform differences between male and female athletes. The three 3D vector-field tests were treated as a single primary family of tests; therefore, α=0.05/3=0.0167 was used as the Bonferroni-corrected significance threshold. Only suprathreshold clusters identified at this corrected threshold were interpreted as confirmatory between-sex differences in 3D GRF.

Building on the overall 3D GRF tests, two-tailed Welch two-sample SPM{t} tests were further applied to the nine scalar components to localize the direction of between-sex differences. Because the nine scalar tests were treated as a single family of directional localization tests, Bonferroni correction was applied using α=0.05/9=0.0056. Positive SPM{t} values indicate higher normalized GRF in male athletes than in female athletes, whereas negative values indicate the opposite pattern. Significant clusters after correction were reported by percentage of the movement cycle, the random-field-theory critical threshold, and cluster-level p values. Uncorrected results were used only as exploratory information on nominal significance and were not used to support the primary conclusions. To assess the sensitivity of parametric SPM inference to distributional assumptions and variance/covariance structure, 5,000 non-parametric permutation tests were performed for each waveform using the same Bonferroni-corrected thresholds as the corresponding parametric analyses: α=0.0167 for the three 3D vector-field tests and α=0.0056 for the nine scalar waveform tests.

### 2.5. Statistical analysis

All data processing and statistical analyses were performed in Python 3.13.7 using custom-written scripts, primarily with NumPy 2.3.5, pandas 2.3.3, spm1d 0.4.53, and Matplotlib 3.10.9. One-dimensional normality tests were conducted on the residuals of the scalar Welch SPM{t} models. The primary inferences for the 3D GRF vector fields and scalar waveforms used Bonferroni-corrected thresholds of α=0.0167 and α=0.0056, respectively. All confirmatory significance statements in the figures and main text were based on SPM inference results at the corresponding corrected thresholds.

## 3. Results

The final analytical sample comprised 44 athletes, including 22 male athletes (height: 177.0 ± 5.2 cm; body mass: 74.4 ± 11.0 kg) and 22 female athletes (height: 169.0 ± 5.9 cm; body mass: 63.8 ± 7.0 kg). After Bonferroni correction, SPM Hotelling’s $T^{\text{2}}$ analyses of the 3D GRF vector fields showed significant between-sex differences in the trailing-foot, lead-foot, and net bilateral GRF among high-level athletes. When the three 3D vector-field tests were treated as a single family of tests (α=0.05/3=0.0167), significant differences were identified in trailing-foot 3D GRF at 26.67%–47.80% (p<0.001) and 64.84%–71.14% (p=0.0108), in lead-foot 3D GRF at 51.03%–61.62% (p=0.0077), and in net bilateral 3D GRF at 54.72%–70.43% (p=0.0014). After further Bonferroni correction of the nine scalar components as a single family of tests (α=0.05/9=0.0056), trailing-foot $F_{x}$ at 62.41%–71.99% (p=0.0017) and $F_{y}$ at 33.98%–47.04% (p<0.001) were higher in male athletes than in female athletes, whereas trailing-foot $F_{z}$ at 42.11%–44.61% was lower in male athletes than in female athletes (p=0.0053). Net bilateral $F_{x}$ at 54.18%–70.41% was higher in male athletes than in female athletes (p<0.001) (Table 1; Figs 2 and 3). No significant suprathreshold clusters survived Bonferroni correction for lead-foot $F_{x}$, $F_{y}$, and $F_{z}$, or for net bilateral $F_{y}$ or $F_{z}$ (see S1 Appendix). At the Bonferroni-corrected thresholds, the sensitivity analyses based on 5,000 permutations reproduced the corresponding parametric confirmatory inference pattern across all 12 waveforms, yielding the same significant and non-significant waveform classifications and the same number of suprathreshold clusters for each waveform.

**Table 1:** Bonferroni-corrected SPM between-sex comparisons of 3D GRF vector fields and scalar components during PiJi in high-level male and female athletes.

Force Component	Critical Threshold (${𝐓}^{2*}$ / ± t*)	Significant Phase (%)	Direction	p-value	Effect size, mean (range)
**Trailing-foot**					
3D GRF	19.879	26.67–47.80	Multivariate difference	<0.001	D = 1.68 (1.35–1.94)
	19.879	64.84–71.14		0.0108	D = 1.45 (1.35–1.50)
F_x_	±3.849	62.41–71.99	Male > Female	0.0017	g = 1.31 (1.16–1.43)
F_y_	±3.839	33.98–47.04	Male > Female	<0.001	g = 1.34 (1.18–1.45)
F_z_	±3.774	42.11–44.61	Male < Female	0.0053	g = −1.12 (−1.13 to –1.12)
**Lead-foot**					
3D GRF	19.224	51.03–61.62	Multivariate difference	0.0077	D = 1.36 (1.33–1.39)
F_x_	±3.815	–	–	–	–
F_y_	±3.724	–	–	–	–
F_z_	±3.741	–	–	–	–
**Net bilateral**					
3D GRF	19.760	54.72–70.43	Multivariate difference	0.0014	D = 1.88 (1.34–2.29)
F_x_	±3.915	54.18–70.41	Male > Female	<0.001	g = 1.77 (1.17–2.17)
F_y_	±3.804	–	–	–	–
F_z_	±3.753	–	–	–	–

Two types of independent-samples SPM tests were used to compare GRF differences between high-level male athletes (n = 22) and female athletes (n = 22) during PiJi. Three-dimensional GRF vector fields were analyzed using Hotelling’s $T^{\text{2}}$ SPM tests, with Bonferroni correction applied across the three 3D tests (α=0.0167). The nine scalar GRF components were analyzed using Welch two-sample SPM{t} tests, with Bonferroni correction applied across the nine tests (α=0.0056). $T^{\text{2}*}$ denotes the random-field-theory critical threshold for the 3D vector-field tests, and $\pm t^{*}$ denotes the two-tailed random-field-theory critical threshold for the scalar components. p denotes the cluster-level probability at the corresponding corrected threshold. The 3D tests were used to determine whether the overall 3D force vector differed between groups; therefore, no Male athletes > Female athletes or Male athletes < Female athletes direction is reported for these tests. Directionality is reported only for scalar components. Effect magnitude was quantified nodewise using Mahalanobis distance (D) for the 3D vector-field comparisons and bias-corrected Hedges’ g for the scalar comparisons. For the 3D comparisons, D was calculated using the pooled within-group covariance matrix of the three force components at each node. For each Bonferroni-significant cluster, the mean effect magnitude and its minimum-to-maximum range across the nodes within that cluster are reported. For scalar comparisons, bias-corrected Hedges’ g values are signed according to the Male athletes – Female athletes contrast; positive and negative values indicate higher values in male and female athletes, respectively. Direction is also reported separately for clarity.

**Fig 2:**
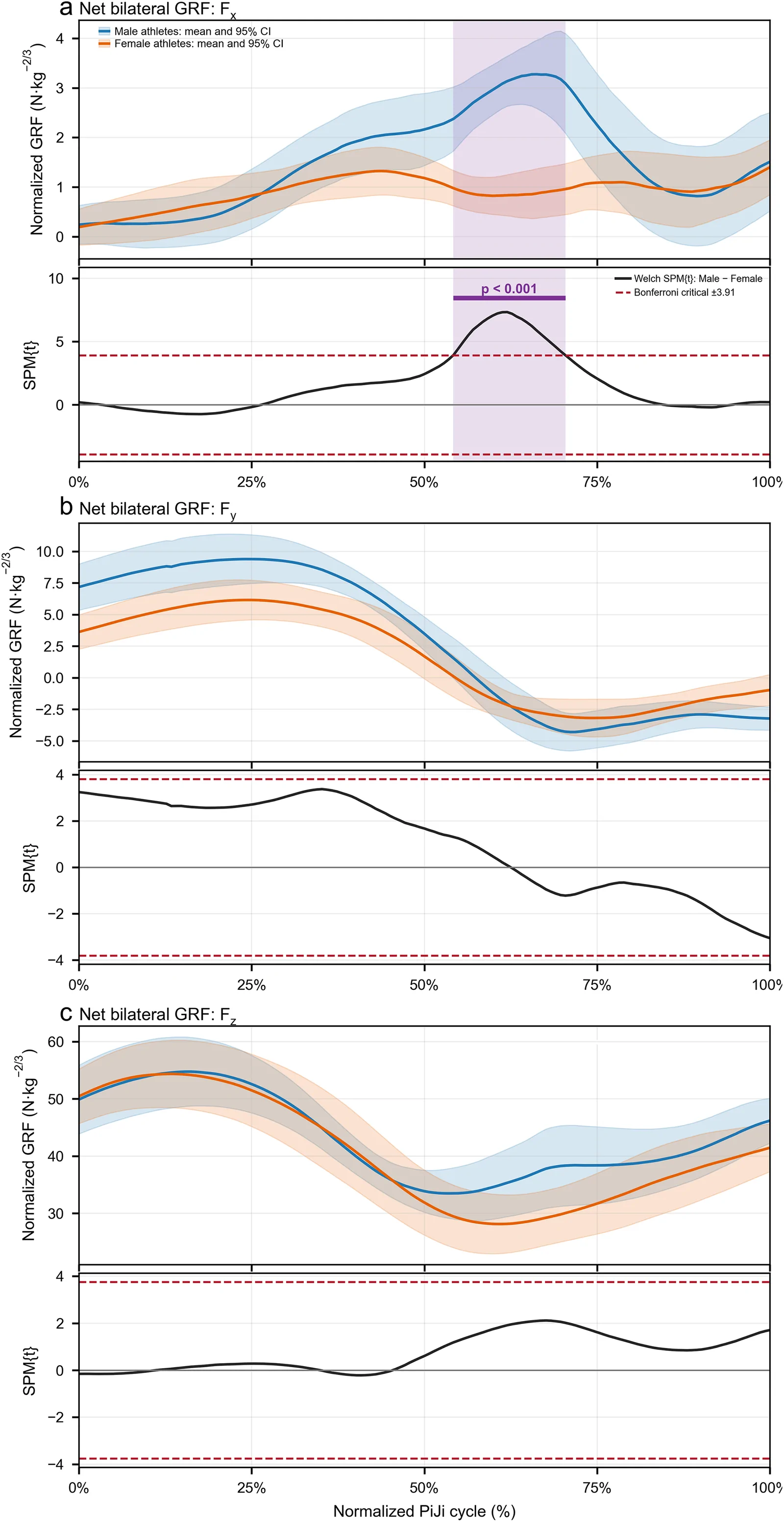
Bonferroni-corrected Welch SPM{t} between-sex comparisons of scalar components of net bilateral 3D GRF during PiJi in high-level Wushu Changbing athletes. Panels a, b, and c show the mediolateral ($F_{x}$), anteroposterior ($F_{y}$), and vertical ($F_{z}$) components of the net bilateral GRF, respectively. In each panel, the upper row shows the group mean and 95% confidence interval for male athletes (blue) and female athletes (orange), and the lower row shows the Welch SPM{t} statistic curve for the contrast “male athletes − female athletes”. The red dashed lines indicate the two-tailed random-field-theory critical thresholds after Bonferroni correction across the nine scalar GRF waveforms (α=0.05/9=0.0056). Purple shading indicates suprathreshold clusters that remained significant after Bonferroni correction; the annotated p values are the corresponding cluster-level probabilities. Uncorrected results are listed only in S1 Appendix and are not used as confirmatory significance evidence in the figure.

**Fig 3:**
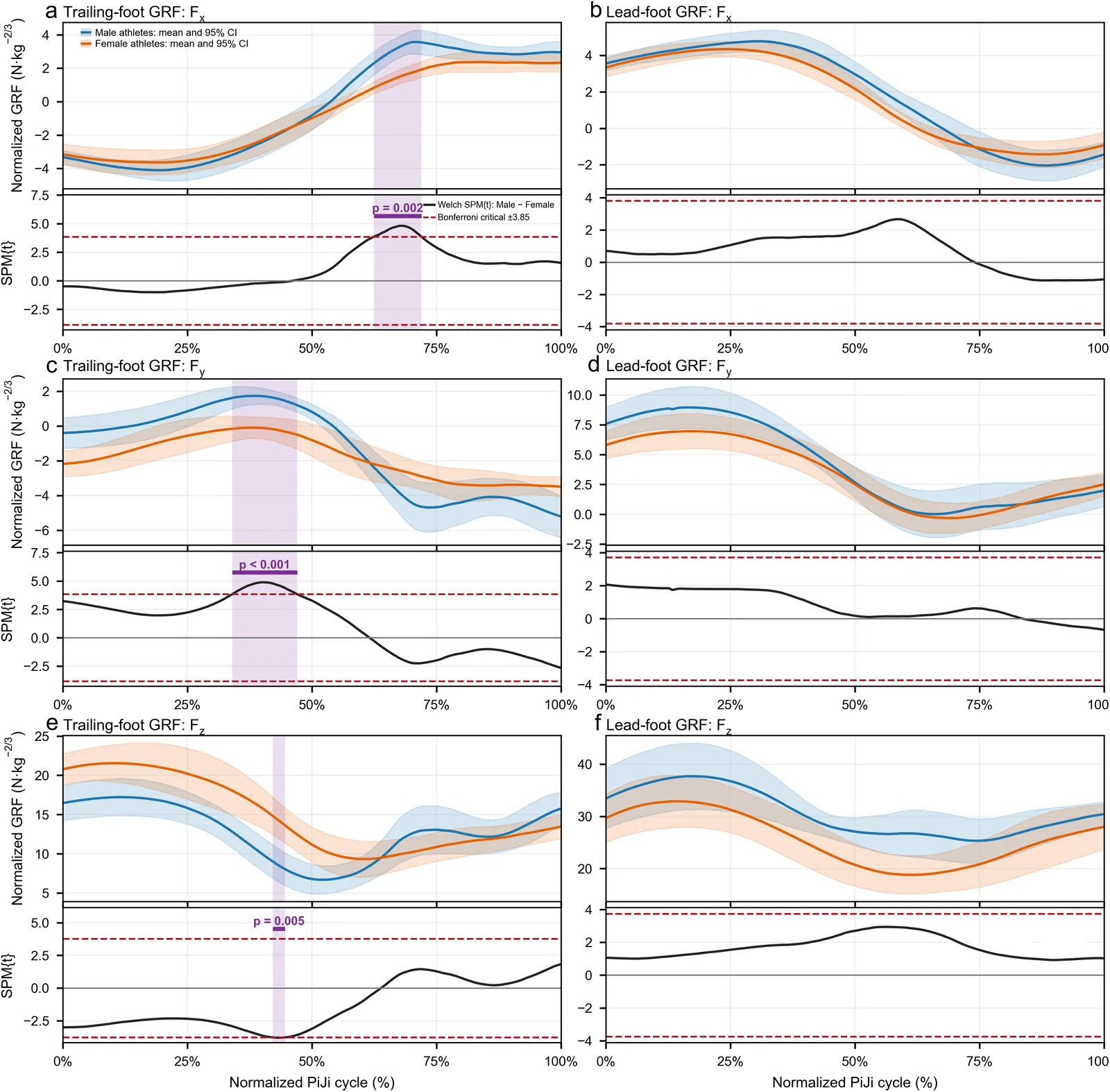
Bonferroni-corrected Welch SPM{t} between-sex comparisons of scalar components of lead-foot and trailing-foot 3D GRF during PiJi in high-level Wushu Changbing athletes. Panels a and b show the mediolateral ($F_{x}$) components of the trailing-foot and lead-foot, respectively; panels c and d show the anteroposterior ($F_{y}$) components of the trailing-foot and lead-foot, respectively; and panels e and f show the vertical ($F_{z}$) components of the trailing-foot and lead-foot, respectively. The significant clusters after Bonferroni correction, shown by purple shading, occurred in trailing-foot $F_{x}$, trailing-foot $F_{y}$, and trailing-foot $F_{z}$. No significant suprathreshold clusters after correction were observed for any lead-foot component.

## 4. Discussion

Using continuous SPM-based comparisons, this study identified phase-dependent between-sex differences in bilateral 3D GRF during PiJi performed with MVE in high-level Wushu Changbing athletes. After Bonferroni correction, the main evidence for between-sex differences was observed in trailing-foot 3D GRF at 26.67%–47.80% (p<0.001) and 64.84%–71.14% (p=0.0108), lead-foot 3D GRF at 51.03%–61.62% (p=0.0077), and net bilateral 3D GRF at 54.72%–70.43% (p=0.0014). Further scalar analyses indicated that the early-to-middle phase difference in trailing-foot 3D GRF mainly reflected higher $F_{y}$ in male athletes, whereas the later-phase difference in trailing-foot 3D GRF mainly reflected higher $F_{x}$ in male athletes; the difference in net bilateral 3D GRF was also primarily associated with higher $F_{x}$ in male athletes. Thus, the between-sex differences did not represent a simple unidirectional increase or decrease in overall force production. Instead, they reflected different patterns of external force organization across feet, directions in the fixed laboratory coordinate system, and movement phases. These findings are consistent with, but do not directly establish, the propulsion-stability strategy trade-off previously proposed by our team using multivariate functional principal component analysis [1,2].

Accordingly, these findings directly addressed the study objective. The hypothesis that bilateral 3D GRF would exhibit movement-cycle-specific sex differences was supported at the vector-field level, as significant differences were observed for the trailing-foot, lead-foot, and net bilateral GRF. At the scalar-component level, support was partial: Bonferroni-corrected directional differences were localized to trailing-foot $F_{x}$, $F_{y}$, and $F_{z}$ and net bilateral $F_{x}$, whereas no individual lead-foot scalar component survived correction. This pattern identifies a between-sex difference in overall lead-foot 3D GRF that was not localized to any single scalar component. Similar studies of implement-based striking have shown that bilateral GRF is jointly influenced by foot position, implement conditions, and individual athlete characteristics [30,31]. Therefore, integrating overall 3D GRF results with Bonferroni-corrected scalar components helps characterize the organization of external lower-limb GRF during PiJi [16].

Net bilateral GRF is an important measure of bilateral coordinated support and global horizontal-plane force regulation [32]. Male athletes showed higher net bilateral $F_{x}$ at 54.18%–70.41% (p<0.001), an interval that strongly overlapped with the difference in net bilateral 3D GRF, suggesting that global mediolateral force regulation during the middle-to-late phase was one of the most robust overall kinetic differences between groups. Mechanically, net bilateral horizontal GRF represents the global horizontal external force acting on the body, and this evidence supports the potential role of horizontal-plane force regulation in the striking kinetic chain [19]. At the lumbopelvic-thoracic level, mediolateral GRF generated through coordinated hip and knee actions can contribute to the whole-body moment about the vertical axis, thereby providing an external mechanical basis for pelvic rotation [19]. This rotating base may stabilize the lumbopelvic-trunk system and support the sequential transfer of mechanical energy from the pelvis through the thorax toward the upper limbs and implement; consistent with this mechanical context, horizontal GRF has been associated with mechanical energy inflow from the hip into the lower torso during batting [5]. Accordingly, the higher middle-to-late net bilateral $F_{x}$ in male athletes may reflect greater reliance on transverse-plane force regulation to rotate and stabilize the pelvis-trunk system as the Changbing descends, rather than simply greater overall force production.

However, because trunk energy transfer was not directly measured in this study, the functional significance of higher net bilateral ($F_{x}$) should be interpreted cautiously and should not be directly equated with greater trunk rotational torque. The partial temporal overlap with the trailing-foot $F_{x}$ difference provides only a compositional clue that the trailing-foot may contribute to this overall external force pattern. Because net bilateral GRF depends simultaneously on the magnitude, direction, and timing of force production from both feet, it cannot be attributed to either foot alone, nor can it be used to infer the causal contribution of a single foot [21,22]. Findings from striking or throwing tasks regarding horizontal GRF, trunk mechanical energy flow, or the functional role of trailing-foot impulse [5,33] may provide hypotheses for future mechanistic research on Wushu Changbing PiJi, but they cannot substitute for joint kinetic evidence in the present study. In addition, golf swing research has shown that the lead-foot is more closely related to the generation of ground reaction moments, whereas the trailing-foot is more closely related to pivoting moments about the vertical axis. This further indicates that foot-specific functional differentiation in fixed-foot striking depends on force direction, point of application, and timing, rather than simply on the peak force produced by a single foot [22]. By contrast, differences in net bilateral $F_{y}$ during the early, middle, and final phases appeared only in the uncorrected results and should therefore be retained as exploratory findings rather than interpreted as confirmatory conclusions.

The trailing-foot results further clarified the potential source of the net bilateral GRF difference and its phase-dependent transition. Male athletes showed higher trailing-foot $F_{y}$ at 33.98%–47.04% (p<0.001), followed by higher trailing-foot $F_{x}$ at 62.41%–71.99% (p=0.0017). This sequential organization, from middle-phase anteroposterior external force regulation to middle-to-late mediolateral external force regulation, suggests that the trailing-foot may contribute to the phase-dependent linkage between body posture control and the downward movement of the implement during PiJi. This pattern should be interpreted as a time-varying reorganization of global external force direction, rather than being directly equated with push-off, braking, or rotational drive in a specific anatomical direction. Trailing-foot $F_{z}$ was higher in female athletes than in male athletes only during a brief interval at 42.11%–44.61% (p=0.0053), which fell within the middle portion of the male-dominant $F_{y}$ difference. Therefore, this finding is more appropriately interpreted as a brief difference in local support-load redistribution or postural stability demands, rather than as evidence for a broad conclusion that female athletes had greater trailing-foot vertical support during both movement initiation and the entire middle phase. Because the same 1.0-kg Changbing represented a greater relative load for female athletes than for male athletes (approximately 1.6% vs. 1.3% of body mass), the brief increase in trailing-foot $F_{z}$ may reflect compensatory vertical support during the downward movement of the implement. However, athlete-implement geometry, implement rotational inertia, and balance were not quantified. Moreover, time normalization limits the ability to distinguish an isolated amplitude difference from an intergroup difference in movement timing. Therefore, this interpretation remains provisional. In baseball pitching, pivot-leg GRF time courses differ across stride conditions and reflect different lower-limb organizations for forward body translation and movement transition [34]; similarly, proximal-to-distal energy transfer depends on time-varying coordinative control [35]. The vertical GRF of the drive leg in softball pitching and lower-limb moment-GRF timing coordination in kendo striking provide relevant background showing that trailing-foot external loading is task- and phase-specific, but they should not be treated as direct evidence for the internal mechanical mechanism underlying PiJi [6,36].

In contrast to the trailing-foot pattern, which showed clear scalar directional differences, the confirmatory lead-foot result was mainly reflected in the overall 3D GRF difference. Lead-foot 3D GRF showed a corrected between-sex difference at 51.03%–61.62% (p=0.0077), but no corrected significant clusters were detected for $F_{x}$, $F_{y}$, or $F_{z}$. Higher lead-foot $F_{z}$ in male athletes appeared only in the uncorrected interval of 53.52%–60.40% (p=0.041) and should therefore be classified as exploratory. The Bonferroni-corrected analyses indicate an overall between-sex difference in lead-foot 3D GRF, without identifying a single scalar component responsible for that difference [37]. Therefore, greater lead-foot vertical loading in male athletes should not be considered a confirmatory conclusion of this study. Previous studies have shown that the lead-foot may contribute to forward body translation control, braking, and distal implement acceleration during rapid implement-based actions [4,15,33,38]. However, the dominant GRF direction differs across tasks; for example, ball speed in the tennis forehand is more strongly associated with peak anteroposterior GRF [39]. In the golf swing, the timing of peak lead-foot GRF, together with trunk and upper-limb velocity timing, is associated with technical proficiency, indicating that interpretation of external loading on the lead-side foot in striking tasks requires integration with trunk, upper-limb, and implement kinematics rather than reliance on a single GRF component alone [40]. Given that golf and baseball swings differ from PiJi in implement-target contact conditions and stance constraints, this evidence provides, at most, a cross-sport comparative context. The present study itself supports only a phase-specific difference in 3D external GRF at the lead-foot and does not determine its specific functional support role.

Overall, the corrected results support a three-level interpretive framework from global to local effects. The middle-to-late difference in net bilateral $F_{x}$ indicates a robust between-sex difference in global mediolateral external force organization. The phase-dependent differences from trailing-foot $F_{y}$ to $F_{x}$, together with the brief $F_{z}$ difference, indicate that this global pattern was accompanied by different temporal distributions of local external force components. In contrast, the lead-foot showed differences only at the overall 3D GRF level, with no corrected significant difference localized to any individual scalar component. SPM preserved the complete GRF waveforms and localized these differences to specific movement phases, thereby addressing the limitations of discrete peak variables in characterizing phase-dependent force-generation features [8,16]. These findings may help identify key foot-specific, global-directional, and phase-dependent features for individualized technical assessment, but their relevance to training design requires sport-specific validation [41,42]. Future studies combining GRF with joint kinematics, electromyography, weapon-tip velocity, and sport-specific performance may further clarify the functional significance of these phase-dependent characteristics [43].

### 4.1. Practical applications

The GRF patterns identified in this study may provide candidate phase-specific variables for future technical-monitoring research on PiJi, rather than validated monitoring indicators. For male athletes, trailing-foot $F_{y}$ during the middle phase (33.98%–47.04%) and trailing-foot and net bilateral GRF $F_{x}$ during the middle-to-late phase (62.41%–71.99% and 54.18%–70.41%, respectively) may be considered candidate waveform-based variables for future evaluation. Their within-athlete reliability, sensitivity to training, and relationships with trunk control, body transfer, or sport-specific performance remain to be established. For female athletes, local phase-specific characteristics of trailing-foot vertical $F_{z}$ during the middle phase may warrant attention, but this finding should not be extended to a training interpretation of consistently greater vertical support from movement initiation through the middle phase. Future lead-foot monitoring research may consider the overall 3D GRF waveform difference at 51.03%–61.62%, rather than presupposing a sex difference in a single $F_{z}$ component. These observations identify candidate priorities for future research rather than validated training recommendations; their relationships with specific training interventions, joint-level mechanisms, and sport-specific outcomes require further evaluation in longitudinal studies.

### 4.2. Limitations

This study has several limitations. The findings are limited to right-hand-dominant, high-level collegiate athletes and may not generalize to other populations. The no-target task omitted implement-target collision and impact buffering, and stance dimensions were not anthropometrically standardized. In addition, the uncovered hard-floor force-plate setup did not reproduce carpeted surface conditions; therefore, its potential influence on horizontal GRF waveforms cannot be excluded. Familiarization volume and duration, as well as self-selected rest intervals, were not recorded; thus, variability in pre-test preparation and recovery, including residual fatigue, could not be quantified, although participants began each trial only after reporting readiness. Although the same readiness-based recovery procedure was applied to both groups, potential group-specific differences in actual rest durations could not be assessed. Finally, sport-specific performance outcomes and direct joint- or muscle-level measures, such as joint kinetics and electromyography, were not included. Therefore, the relationships between the observed GRF differences, PiJi performance, and their underlying neuromuscular mechanisms remain unresolved.

## 5. Conclusion

Using continuous phase-based SPM analysis, this study revealed sex-related differences in bilateral 3D GRF during PiJi performed with MVE in high-level Wushu Changbing athletes. After Bonferroni correction, the 3D vector fields of the trailing-foot, lead-foot, and net bilateral GRF showed phase-specific between-sex differences. Scalar localization showed that male athletes had higher trailing-foot $F_{y}$ during the middle phase (33.98%–47.04%) and higher trailing-foot $F_{x}$ (62.41%–71.99%) and net bilateral $F_{x}$ (54.18%–70.41%) during the middle-to-late phase; female athletes showed only a brief period of higher trailing-foot vertical $F_{z}$ during the middle phase (42.11%–44.61%). In contrast, the confirmatory evidence for lead-foot GRF was mainly reflected in the overall 3D waveform difference and was not further localized to a specific scalar component. These findings indicate that sex-related GRF differences during high-level PiJi vary across feet, force directions, and movement phases. This evidence may inform future research on external-GRF-based monitoring and training evaluation during key movement phases, but its specific functional significance requires further validation in future studies integrating kinematics, muscle activity, and technical performance indicators. Many unarmed and weapon-based techniques in traditional Chinese Wushu, including those of the Ma-style Tongbei Wushu school, remain insufficiently explained in modern biomechanical terms, which may contribute to their perceived mystique among audiences from other cultural backgrounds. This research series represents an initial attempt to quantify these techniques, with further studies planned to elucidate their underlying biomechanical organization and provide a modern scientific pathway for the development, cross-cultural exchange, and transmission of traditional Chinese Wushu.

## Supporting information

S1 Appendix SPM between-sex comparison results without Bonferroni correction. (DOCX)

S1 Data Anonymized participant-level data for the SPM analyses. The CSV file contains allometrically normalized bilateral and net 3D GRF waveforms from 44 athletes across the normalized PiJi cycle.
(CSV)
