# Supplementary material for: Statistical Parametric Mapping reveals sex-specific, phase-dependent differences in bilateral three-dimensional ground reaction force during Wushu Changbing PiJi in high-level athletes
Source: PLoS One. 2026 Nov 4;21(11):e0359797. doi: 10.1371/journal.pone.0359797 (PMC13734589; doi:10.1371/journal.pone.0359797)
Supplement: Supplementary file 1 [file pone.0359797.s001.docx]

Appendix 1. SPM between-sex comparison results without Bonferroni correction

| **Force Component** | **Critical Threshold (**$\mathbf{T}^{\mathbf{2*}}$**/**$\mathbf{t}^{\mathbf{*}}$**)** | **Significant Phase (%)** | **Direction** | **p-value** |
| --- | --- | --- | --- | --- |
| Trailing-foot | | | | |
| 3D GRF | 16.014 | 21.69–49.08 | Multivariate difference | <0.001 |
|  |  | 62.59–72.32 |  | 0.022 |
| Fx | ±3.009 | 59.29–74.64 | Male > Female | 0.007 |
| Fy | ±2.998 | 0–3.7 | Male > Female | 0.045 |
|  |  | 30.40–51.17 | Male > Female | 0.002 |
| Fz | ±2.924 | 0–5.25 | Male < Female | 0.043 |
|  |  | 34.03–50.94 | Male < Female | 0.011 |
| Lead-foot | | | | |
| 3D GRF | 15.389 | 47.19–64.16 | Multivariate difference | 0.011 |
| Fx | ±2.965 | — | — | >0.05 |
| Fy | ±2.863 | — | — | >0.05 |
| Fz | ±2.886 | 53.52–60.40 | Male > Female | 0.041 |
| Net bilateral | | | | |
| 3D GRF | 15.900 | 0.00–5.05 | Multivariate difference | 0.041 |
|  |  | 35.41–43.54 |  | 0.030 |
|  |  | 53.99–71.39 |  | 0.005 |
|  |  | 98.42–100.00 |  | 0.049 |
| Fx | ±3.063 | 52.21–72.34 | Male > Female | <0.001 |
| Fy | ±2.957 | 0.00–7.73 | Male > Female | 0.035 |
|  |  | 28.93–40.21 | Male > Female | 0.023 |
|  |  | 99.02–100 | Male < Female | 0.0497 |
| Fz | ±2.899 | — | — | >0.05 |
